# Dysregulation of the Sirt5/IDH2 axis contributes to sunitinib resistance in human renal cancer cells

**DOI:** 10.1002/2211-5463.13090

**Published:** 2021-02-08

**Authors:** Liang Meng, Deqiang Chen, Gaopei Meng, Li Lu, Chenggang Han

**Affiliations:** ^1^ Department of Computer Tomography Cangzhou Central Hospital China

**Keywords:** clear cell renal cell carcinoma, desuccinylation, isocitrate dehydrogenase 2, Sirt5, sunitinib

## Abstract

Sunitinib (Sun), a tyrosine kinase inhibitor of vascular endothelial growth factor receptor, is the standard first‐line treatment against advanced clear cell renal cell carcinoma (RCC), but resistance to therapy is inevitable. Reactive oxygen species production is associated with sensitivity to chemotherapy, but the underlying mechanisms are not completely understood. Here, we investigated the mechanisms contributing to Sun resistance using the RCC cell lines ACHN and 786‐O. We report that Sun‐resistant cells exhibited reduced apoptosis, increased cell viability, increased reactive oxygen species production and disrupted mitochondrial function. Furthermore, chronic Sun treatment resulted in an up‐regulation of Sirt5/isocitrate dehydrogenase 2 (IDH2) expression levels. Knockdown of Sirt5/IDH2 impaired mitochondrial function and partially attenuated Sun resistance. Finally, up‐regulation of Sirt5 enhanced the expression of IDH2 via modulation of succinylation at K413 and promoted protein stability. In conclusion, dysregulation of Sirt5/IDH2 partially contributes to Sun resistance in RCC cells by affecting antioxidant capacity.

## Graphical abstract

Sunitinib‐resistant cells exhibited up‐regulation of Sirt5/isocitrate dehydrogenase 2 (IDH2) expression levels, which confer drug resistance. Up‐regulation of Sirt5 enhanced the expression of IDH2 via modulation of succinylation at K413 and promotion of protein stability. Dysregulation of Sirt5/IDH2 partially contributes to Sun resistance by disturbing mitochondrial functions and affecting antioxidant capacity of renal cell carcinoma cells.

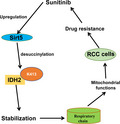

**Abbreviations**
ccRCC: clear cell renal cell carcinoma
CHX: cyclohexane
CI+II_ETS_: the noncoupled state complex I+II supported noncoupled respiration
CI+II_OXPHOS_: maximal oxidative phosphorylation
CI_OXPHOS_: complex I‐dependent oxidative phosphorylation
CCK‐8: Cell Counting Kit 8
COX‐IV: cytochrome c oxidase IV
DHE: dihydroethidium
ECAR: extracellular acidification rate
GSH: glutathione
IDH2: isocitrate dehydrogenase 2
PDHE1α, pyruvate dehydrogenase E1α subunit; PI, propidium iodide; R: routine respiration
ROS: reactive oxygen species
SDH: succinate dehydrogenase
SEM: standard error of the mean
sirt: sirtuin
Sun‐R: sunitinib resistant

Renal cancer has been reported to be the seventh most common cancer in the world, with an alarming increase in morbidity and mortality in recent years, while ~70–80% of all renal cell carcinoma (RCC) histological subtypes is clear cell RCC (ccRCC) [1, 2]. However, most of the patients with RCC are diagnosed with metastatic RCC when first enrolled in the hospital and require systemic therapies [3]. Recently, several new agents of targeted therapeutics have been demonstrated to be effective in patients with advanced ccRCC, characterized by therapies against vascular endothelial growth factor and mammalian target of rapamycin [4]. Among them, sunitinib is a broad‐spectrum inhibitor of receptor tyrosine kinases that serves as the standard of treatment for first‐line therapy of advanced ccRCC [5]. Although there is an increased trend of improved survival, not all patients with RCC respond to sunitinib, and most of the patients experience chemoresistance at last [6, 7].

The mechanisms of chemoresistance to sunitinib are not fully understood and have been variably reported [8, 9]. Understanding the mechanism of drug resistance may not only guide subsequent treatment selection but may also provide insights into optimal upfront combination treatment approaches. Cell metabolism alteration and antioxidant capacity have been reported to be strictly correlated with chemoresistance to various tumors, but for sunitinib resistance, the studies are few [10, 11].

Previous studies indicated that mitochondrial functions play important roles in the tumorigenesis, cancer progression and chemotherapies [12]. Cancer cells exhibited mitochondrial metabolism alterations that support cancer cell proliferation, survival and/or progression and include up‐regulation of oxidative metabolism and use of alternative substrates, oncometabolites, increased superoxide production, mutated mitochondrial DNA and altered mitochondrial morphology and dynamics [12]. The sirtuins (Sirts) are a family of orthologues that share extensive homologies with the silent information regulator 2 gene in yeast [13]. In mammals, Sirts comprise seven members, termed SIRT1–SIRT7, respectively, and are important in the regulation of cancer metabolism, cellular proliferation, aging, survival and oncogenesis or tumor suppression [14], even chemoresistance [15]. Among them, Sirt5 has been demonstrated to be located in the mitochondria and to modulate mitochondrial functions by regulating posttranslational patterns by removing succinyl, malonyl and glutaryl groups from protein targets within the mitochondrial matrix [16]. However, the role of mitochondrial reprogramming by Sirt5 in the chemoresistance of sunitinib in RCC has not been defined. In this study, the sunitinib‐resistant (Sun‐R) RCC cells were used, and the concrete mechanisms of Sirt5‐induced mitochondrial changes have been investigated.

## Results

### Sun‐R renal cancer cells exhibited altered metabolic features

**Fig. 1:**
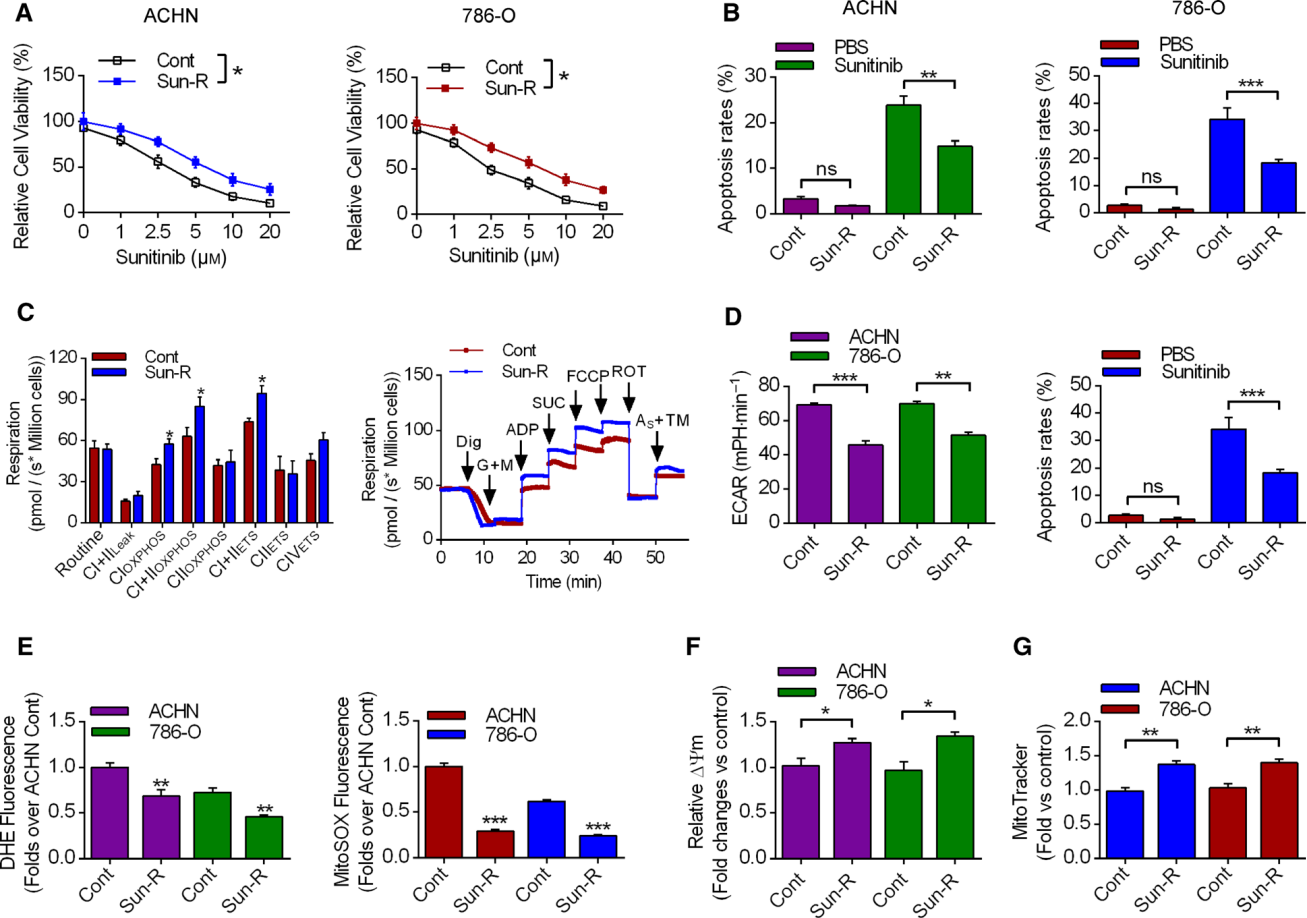
The metabolic features of Sun‐R renal cancer cells. (A) Cell viability assay was conducted to analyze the sensitivity of RCC cells to sunitinib. *n* = 3. (B) The apoptosis rates of RCC cells were analyzed by flow cytometry treated in the presence or absence of 10 μm sunitinib. *n* = 3. (C, D) High‐resolution respirometry was conducted to analyze the metabolic features of Sun‐R cells by analyzing the respiration, ECAR and lactate secretions. *n* = 3. (E) The levels of cellular and mitochondrial ROS production were measured by specific dyes in both ACHN and 786‐O cells. *n* = 3. (F, G) The levels of mitochondrial membrane potential and MitoTracker fluorescence were measured, with fold changes calculated with the control group. *n*=3. All data were presented as mean ± SEM; *P < 0.05, **P < 0.01, ***P < 0.001. Unpaired Student's *t*‐test was used to determine statistical significance.

Firstly, Sun‐R renal cancer cells (ACHN and 786‐O) were produced by chronic incubation with low concentration of sunitinib; then the cell viability assay was performed in these two cell lines. Figure 1A showed that Sun‐R cells exhibited increased cell viability compared with the control group in both ACHN and 786‐O cells. Meanwhile, Sun‐R cells had a lower apoptotic ratio when treated with sunitinib, compared with normal cells (Fig. 1B). Furthermore, the mitochondrial metabolic status was analyzed to better illustrate the mechanisms of Sun‐R cells. High‐resolution respirometry results showed that Sun‐R cells had increased complex I‐dependent oxidative phosphorylation (CI_OXPHOS_), maximal oxidative phosphorylation (CI+II_OXPHOS_) and the noncoupled state complex I+II supported noncoupled respiration (CI+II_ETS_) values (Fig. 1C). Moreover, we analyzed the extracellular acidification rate (ECAR) values and lactate secretion in both control and Sun‐R cells, showing that Sun‐R cells had decreased ECAR values and lactate secretions compared with the control group (Fig. 1D), indicating that Sun‐R cells had increased efficiency in cell energy metabolism. Also, cellular reactive oxygen species (ROS) and mitochondrial‐specific ROS levels were all decreased in Sun‐R cells (Fig. 1E). The mitochondrial membrane potentials and mitochondrial contents were measured, showing that Sun‐R cells exhibited increased membrane potentials and content (Fig. 1F,G). These results indicated that Sun‐R cells had improved mitochondrial functions and decreased ROS productions.

### Sirt5 up‐regulation participates in the chemoresistance of sunitinib

**Fig. 2:**
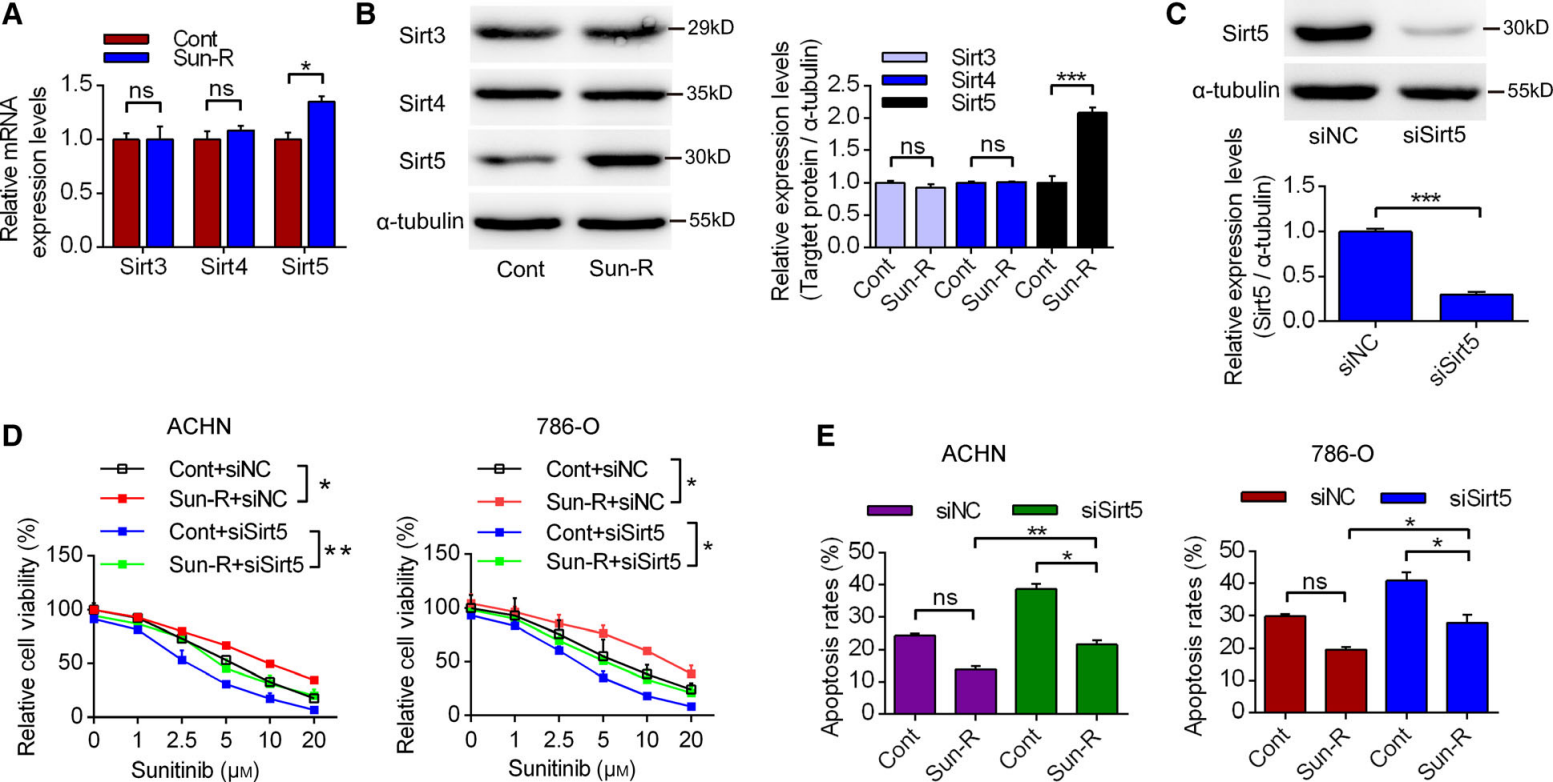
The effects of sirt5 on the chemoresistance in renal cancer cells. (A, B) The expression profiles of Sirt3, Sirt4 and Sirt5 mRNA and protein are presented. *n* = 3. (C) The effects of Sirt5‐specific siRNA on the expression levels of Sirt5, detected by western blotting using α‐tubulin as a loading control. *n* = 3. (D) Cell viability assay to analyze the sensitivities of RCC cells to sunitinib upon siNC or siSirt5 treatment. *n* = 3. (E) The apoptosis rates of RCC cells were analyzed by flow cytometry treated with siNC or siSirt5. *n* = 3. The error bars indicate SEM. *P < 0.05, **P < 0.01, ***P < 0.001. Unpaired Student's *t*‐test and one‐way ANOVA followed by Bonferroni *post hoc* tests were used to determine statistical significance.

Sirt3, Sirt4 and Sirt5 are all distributed in the mitochondria and are important regulators of mitochondrial functions. It was shown that the mRNA expression profiles of Sirt3 and Sirt4 did not change significantly, whereas Sirt5 mRNA levels were up‐regulated in Sun‐R cells (Fig. 2A). However, immunoblotting showed that the expression of Sirt5 increased significantly in Sun‐R cells compared with control cells, whereas the expression levels of Sirt3 and Sirt4 did not change significantly (Fig. 2B). Using Sirt5‐specific siRNA, it was shown that it could efficiently decrease the expression levels of Sirt5 in Fig. 2C. Meanwhile, it was demonstrated that Sirt5 knockdown could partially decrease the cell viability and increase the apoptosis rates, compared with the si‐Negative control (siNC) group, upon treatment with sunitinib (Fig. 2D,E). Therefore, these results suggest that Sirt5 up‐regulation is partially responsible for the chemoresistance of sunitinib in RCC cells.

### Sirt5 improved mitochondrial metabolism via up‐regulating the antioxidant enzymes

**Fig. 3:**
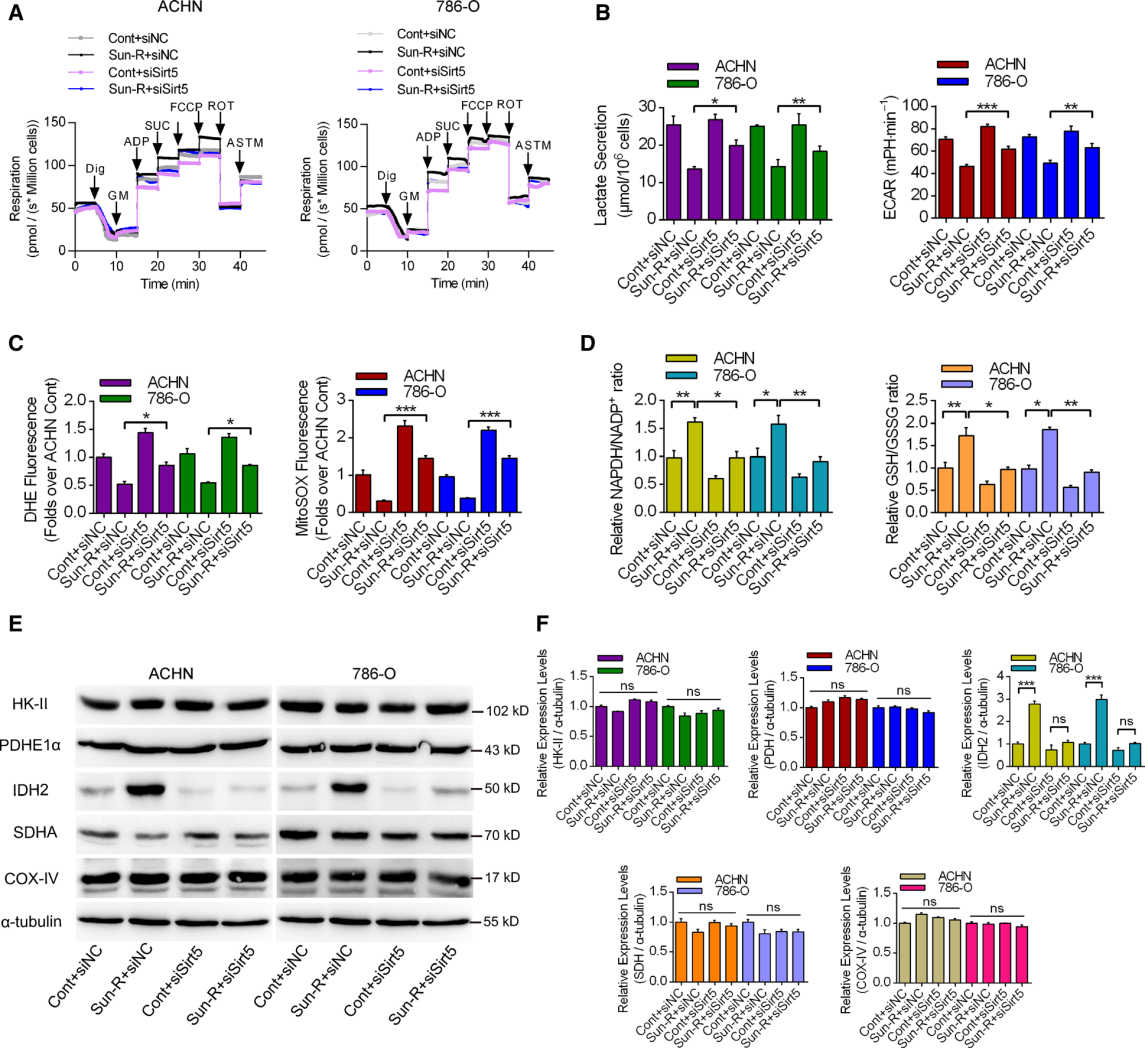
The effects of sirt5 knockdown on mitochondrial respiratory functions. (A, B) High‐resolution respirometry experiments to analyze the metabolic features of RCC cells, treated with siNC or siSirt5, by analyzing the respiration, ECAR and lactate secretions. Dig, digitonin; GM, glutamate and malate; ADP, adenosine diphosphate; SUC, succinate; FCCP, 2‐[[4‐(trifluoromethoxy)phenyl]hydrazinylidene]propanedinitrile; ROT, rotenone; ASTM, ascorbate sodium salt and TMPD; *n* = 3. (C) The cellular and mitochondrial ROS productions were measured by dihydroethidium (DHE) and MitoSOX, treated with siNC or siSirt5, in both ACHN and 786‐O cells. *n* = 3. (D) The NADPH/NADP^+^ and GSH/GSSG ratios were analyzed in RCC cells with the same treatments in (C). *n* = 3. (E, F) The expression levels of key enzymes in the metabolic pathway in RCC cells treated with siNC or siSirt5 in both control and Sun‐R cells by western blotting, using α‐tubulin as a loading control. Then three repeated immunoblotting bands were quantified and analyzed in (F). *n* = 3. The error bars indicate SEM. *P < 0.05, **P < 0.01, ***P < 0.001. Unpaired Student's *t*‐test and one‐way ANOVA followed by Bonferroni’s *post hoc* tests were used to determine statistical significance.

Firstly, we measured the effects of Sirt5 knockdown on mitochondrial respiratory functions by high‐resolution respirometry. Figure 3A showed that siSirt5 significantly decreased complex I_OXPHOS_, complex I+II_OXPHOS_ and complex I+II_ETS_ in both ACHN and 786‐O Sun‐R cells compared with siNC. Meanwhile, the lactate secretions and ECAR rates were also detected upon Sirt5 knockdown, elucidating that Sun‐R cells exhibit decreased glycolysis with decreased ECAR rates and lactate secretions, whereas Sirt5 knockdown partially increased these values (Fig. 3B). Also, Sirt5 knockdown could also increase the levels of both cellular ROS and mitochondrial‐specific ROS in both ACHN and 786‐O cells (Fig. 3C). Furthermore, the NADPH/NADP^+^ ratio and the glutathione (GSH)/oxidized glutathione (GSSG) ratio were all increased in Sun‐R cells, with Sirt5 knockdown decreasing the levels of these ratios, showing that modulation of Sirt5 levels significantly influenced the antioxidant capacity of RCC cells (Fig. 3D). To systematically investigate the target of Sirt5, we analyzed the expression profiles of key redox‐regulatory enzymes by western blotting. It was demonstrated that the expression levels of hexokinase II, pyruvate dehydrogenase E1α subunit (PDHE1α), succinate dehydrogenase (SDH) and cytochrome c oxidase IV (COX‐IV) did not change significantly, while Sun‐R cells exhibited up‐regulated isocitrate dehydrogenase 2 (IDH2) levels, which could be attenuated by Sirt5 knockdown (Fig. 3E,F). These results demonstrated that siSirt5 significantly decreased the antioxidant capacity in Sun‐R cells, possibly by up‐regulating IDH2 levels.

### The effects of IDH2 knockdown on sunitinib resistance in RCC cells

**Fig. 4:**
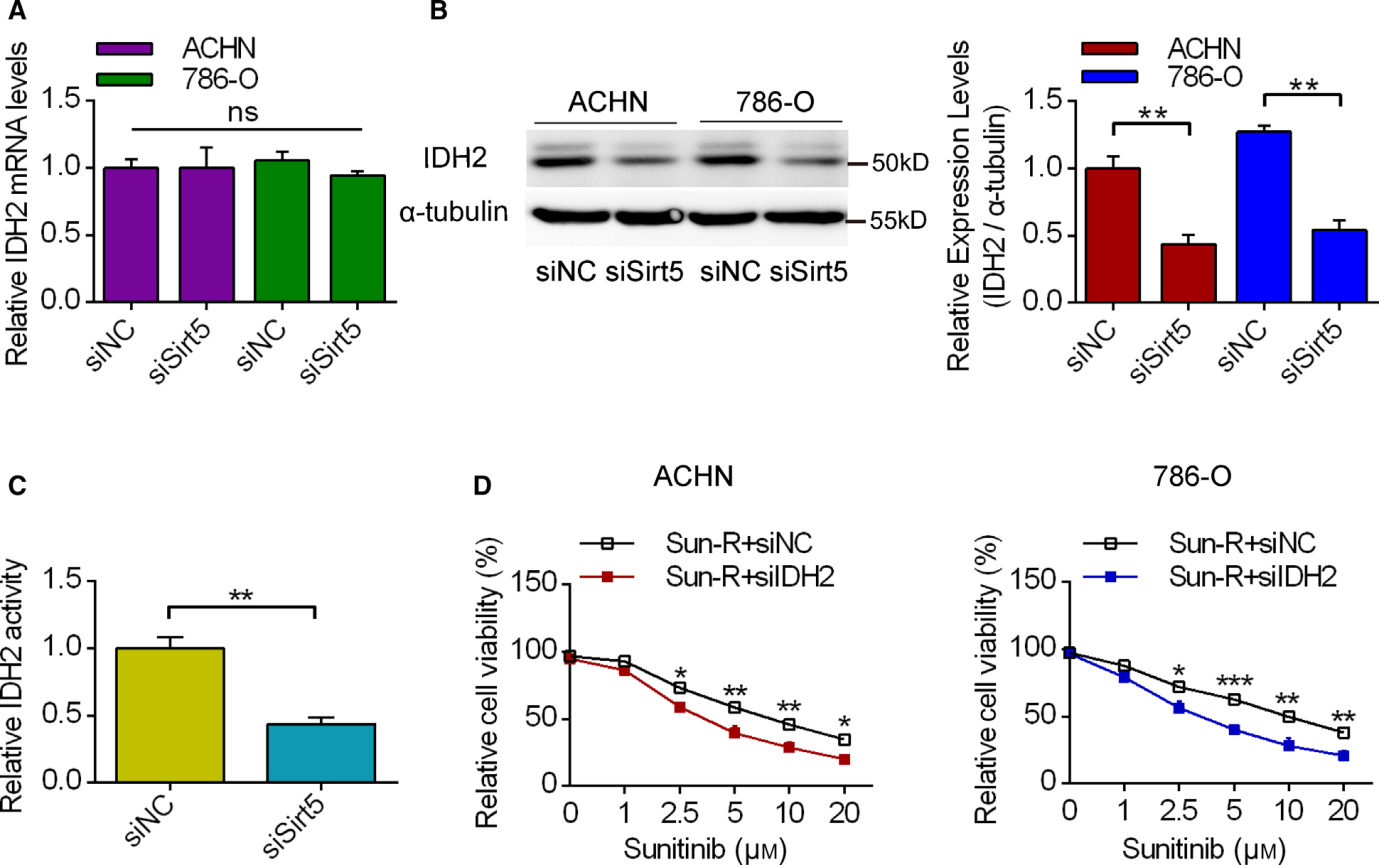
The effects of IDH2 knockdown on sunitinib resistance. (A, B) Both mRNA and protein expression levels of IDH2 were analyzed in RCC cells, treated by siNC or siSirt5. *n* = 3. (C) The effects of siNC and siSirt5 on IDH2 activities in HEK293T cells. *n* = 3. (D) The effects of siNC/siIDH2 on cell viability upon sunitinib resistance in Sun‐R RCC cells. *n* = 3. The error bars indicate SEM. *P < 0.05, **P < 0.01, ***P < 0.001. Unpaired Student's *t*‐test and one‐way ANOVA followed by Bonferroni’s *post hoc* tests were used to determine statistical significance.

To deeply reveal the mechanisms of IDH2 levels by Sirt5, we first analyzed IDH2 mRNA and protein levels upon Sirt5 knockdown. It was shown that Sirt5 knockdown did not affect the IDH2 mRNA level in both ACHN and 786‐O cells (Fig. 4A). For protein levels, it was shown that Sirt5 knockdown significantly decreased the IDH2 levels in both ACHN and 786‐O cells (Fig. 4B). Moreover, Sirt5 knockdown significantly inhibited the IDH2 activities (Fig. 4C). Using cell viability assay, it was shown that IDH2 knockdown by specific siRNA significantly inhibited the viable cells upon sunitinib treatment compared with siNC (Fig. 4D). These results indicated that Sirt5/IDH2 might become an important mechanism for drug resistance.

### Sirt5 enhanced the protein stability of IDH2 by promoting posttranslational desuccinylation

**Fig. 5:**
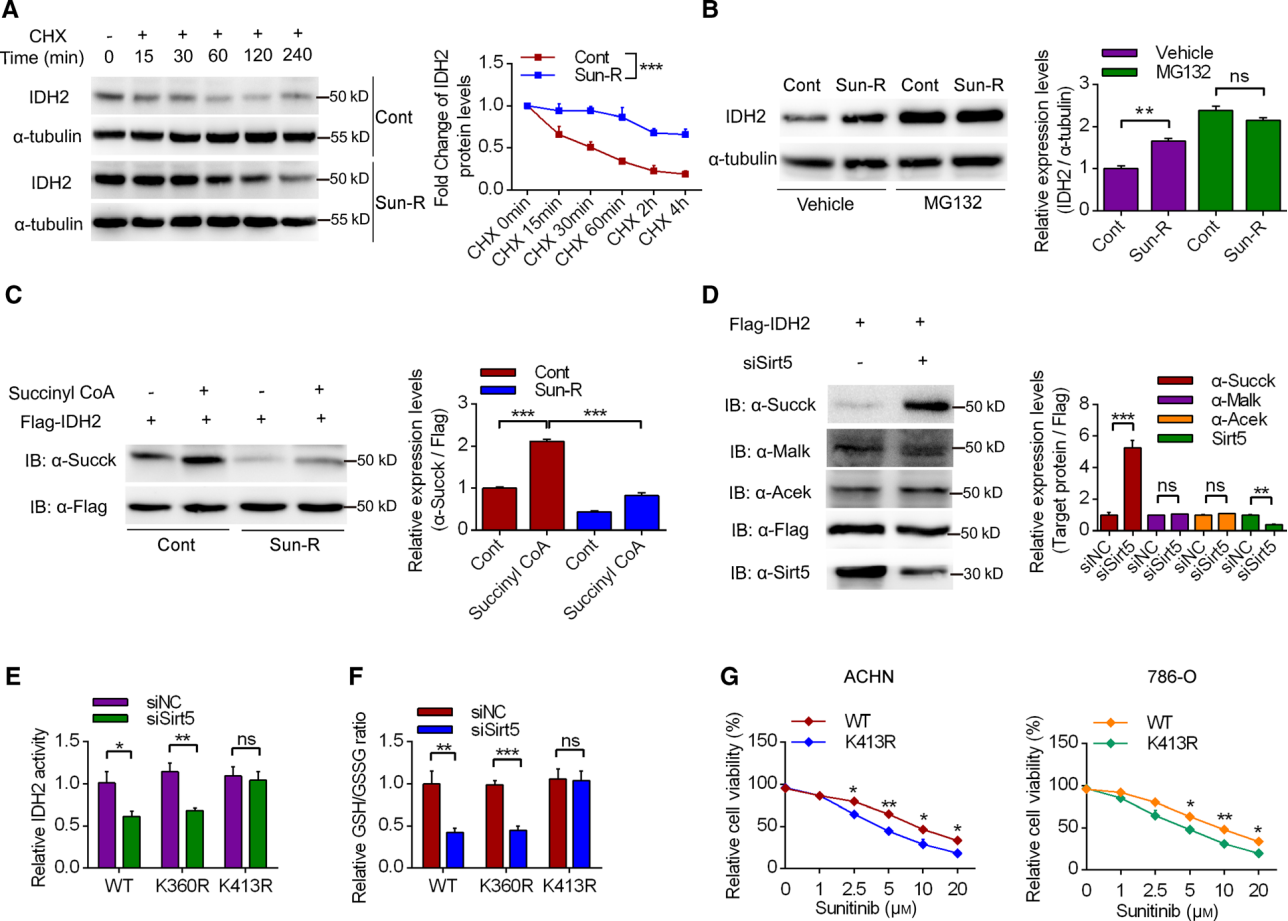
The mechanisms of sirt5 in regulating the expression levels of IDH2. (A) The protein stability assay was conducted in Sun‐R cells by using CHX in a time‐dependent manner. *n* = 3. (B) The protein stability assay was conducted in Sun‐R cells by using 10 μm MG132. *n* = 3. (C, D) The expression of IDH2 was analyzed by western blotting in RCC cells, treated with succinyl CoA or siNC/siSirt5. (E, F) The IDH2 enzymic activity and GSH/GSSG ratio were measured in HEK‐293T cells, transfected with WT, K360R and K413R mutant plasmids. *n* = 3. (G) The effects of WT and K413R mutant IDH2 on sunitinib resistance in RCC cells using CCK‐8 assay. *n* = 3. The error bars indicate SEM. *P < 0.05, **P < 0.01, ***P < 0.001. Unpaired Student's *t*‐test and one‐way ANOVA followed by Bonferroni *post hoc* tests were used to determine statistical significance.

To deeply analyze the mechanism of Sirt5 in regulation of IDH2 expression, we analyzed the protein stabilities by inhibiting protein synthesis by using cyclohexane (CHX), a protein synthesis inhibitor. It was shown in Fig. 5A that Sun‐R cells exhibited enhanced IDH2 protein stabilities compared with the control group. Meanwhile, inhibition of protein degradation by supplementation of MG132 showed that Sirt5 up‐regulation in Sun‐R cells was mediated by enhancing protein stabilities (Fig. 5B). Furthermore, previous proteomic studies have identified a large number of succinylated, malonylated and glutarylated proteins that could be modified by Sirt5 [17, 18, 19]. It has previously been reported that succinyl‐CoA can lead to nonenzymatic succinylation of lysine residues in proteins [20]. As expected, when immunoprecipitated Flag‐tagged IDH2 was incubated with succinyl‐CoA *in* *vitro*, its lysine succinylation was dramatically increased, whereas the IDH2 protein in Sun‐R cells exhibited decreased levels of succinylation in the absence of succinyl‐CoA (Fig. 5C). Furthermore, co‐overexpression of Flag‐IDH2 with siSIRT5 enhanced IDH2 succinylation, whereas it did not affect lysine glutarylation, malonylation and acetylation of Flag‐IDH2 (Fig. 5D). For the detailed succinylation sites of IDH2, it was reported that 17 different lysine residues in IDH2 can be potentially modified by succinylation [17, 18, 21]. Among these lysine residues, several sites seem to be highly targeted by SIRT5, including K80, K155, K263, K360 and K413. Based on the crystal structure [22], two lysine sites, K360 and K413, which are adjacent to the substrate binding site, imply that succinylation of these two residues might compromise the binding pocket for isocitrate and inhibit IDH2 activity. In Fig. 5E,F, we have further investigated the role of succinylations in K360 and K413 residues using a mutation of each of these two putative succinylation sites to arginine (R) (K360R and K413R). It was shown that the K413 mutation did not significantly affect the IDH2 activity and GSH/GSSG ratio upon Sirt5 knockdown. More importantly, the K413R mutation could significantly sensitize RCC cells to sunitinib treatment using CCK‐8 assay (Fig. 5G).

## Discussion

A better understanding of the molecular basis of RCC has led to introduction of antiangiogenic therapies for this tumor, although these drugs yield partial responses in a minority of patients with no evidence of complete responses [23, 24]. Elucidating the mechanisms of RCC resistance to sunitinib has been attached vital importance. Several hypotheses have been proposed regarding the mechanism underlying resistance to sunitinib [25, 26], such as the activation and/or up‐regulation of other proangiogenic signaling pathways [27], recruitment and survival of myeloid‐derived suppressor cells with sustained immune suppression and angiogenesis [28], increased tumor cell invasiveness to escape from oxygen and nutrition deprivation [29]. In this study, we showed that Sun‐R cells exhibited resistance to sunitinib, with less apoptosis and increased viability. Meanwhile, improved mitochondrial metabolism and decreased ROS levels were present in Sun‐R cells. Furthermore, we showed that chronic sunitinib treatment up‐regulated Sirt5/IDH2 expression levels, whereas knockdown of Sirt5/IDH2 partially attenuated sunitinib resistance. At last, Sirt5 up‐regulation promoted the expression of IDH2 via modulating its succinylation at K413 residue and promoting protein stabilities. RCC is now characterized by a reprogramming of energetic metabolism, and mutations in target genes were involved in metabolic pathways. In particular, the metabolic flux through glycolysis is partitioned [30, 31], and mitochondrial bioenergetics and OXPHOS are impaired, as well as lipid metabolism [32]. In this scenario, it has been shown that Sirt5 targets the pyruvate dehydrogenase complex and SDH to suppress their biochemical activities and mitochondrial respiration driven by these complexes [17, 33].

Recent studies indicated that oxidative stress was involved in the drug resistance in renal cancer. For example, Ponnusamy *et al*. [34] reported that chronic oxidative stress increases resistance to doxorubicin‐induced cytotoxicity in renal carcinoma. Meanwhile, imbalance of oxidative stress also contributed to chemoresistance in renal cancer [35]. Furthermore, altered mitochondrial metabolism also has been shown to regulate drug sensitivities, especially in targeted therapy [10, 36]. In our study, we showed that Sun‐R cells exhibited decreased ROS productions and enhanced mitochondrial metabolism, which help cancer cells better adapt to the environment and better elucidate the mechanism of sunitinib resistance.

Sirt5 possesses a mitochondrial localization sequence, with a majority of Sirt5 protein localized in the mitochondrial matrix [37]. Regarding the expression patterns of Sirt5 in cancer, it has been demonstrated that Sirt5 mRNA expression tends to be elevated in tumor tissues compared with the corresponding normal tissues [38, 39]. For example, it has been demonstrated that the Sirt5 mRNA and protein expression levels are frequently elevated in human lung cancers [39]. Also, Sirt5 mRNA expression levels were increased in invasive breast tumors [38]. However, the expression patterns of Sirt5 in RCC have not been reported in previous studies, and thus require further investigation. Regarding the role of Sirt5 in regulating cancer cell metabolism, Bringman‐Rodenbarger *et al*. [16] presented a comprehensive and elaborate review. Sirt5 has been elucidated to promote the glycolysis by demalonylating glyceraldehyde‐3 phosphate dehydrogenase and other enzymes in the glycolytic cascade in mouse liver, enhancing the glycolytic flux [19]. In our study, we showed that Sun‐R cells had decreased glycolysis compared with normal ones, whereas the expression levels of PDH have not changed significantly, indicating that other key enzymes might experience some modifications.

IDH2 is the mitochondrial NADPH‐dependent enzyme that catalyzes the conversion of isocitrate to α‐ketoglutarate by oxidative decarboxylation. NADPH produced in this process is a fundamental cofactor of GSH‐associated mitochondrial antioxidant defense systems, including GSH peroxidase and GSH reductase [40], which maintain the availability of GSH in a reduced state in the mitochondria. Therefore, IDH2 is crucial for GSH turnover and defense against ROS. In cancer metabolism, the role of IDH2 has been reported to guard the redox balance in the cancer cells. For instance, IDH2 is a dual regulator of cancer bioenergetics and tumor cell motility, which reprograms mitochondrial dynamics to differentially adjust energy production or promote tumor cell invasion in response to microenvironment conditions [41]. Meanwhile, down‐regulation of IDH2 sensitizes cancer cells to erastin‐induced ferroptosis, which might become a new strategy in cancer chemotherapy [42]. In this study, we demonstrated that the IDH2 expression levels positively correlated with the Sirt5 expressions in Sun‐R RCC cells, while Sirt5 promoted the protein stability of IDH2 by promoting desuccinylation. Also, knockdown of the Sirt5/IDH2 pathway could become a new strategy in alleviating sunitinib resistance.

## Conclusions

This study indicates that Sirt5/IDH2 up‐regulation is involved in Sun‐R RCC cells via enhancing the mitochondrial metabolism and the antioxidant capacity. Targeting the Sirt5/IDH2 pathway could become a new strategy in alleviating sunitinib resistance.

## Materials and methods

### Cell culture and treatment

RCC cell lines were obtained from the American Type Culture Collection (Rockville, MD, USA). ACHN was cultured in Dulbecco's modified Eagle's medium (DMEM), while 786‐O was propagated in RPMI‐1640 (Life Technologies, Carlsbad, CA, USA), supplemented with 10% FBS, 100 U·mL^−1^ penicillin and 100 μg·mL^−1^ streptomycin. The culture environment was 95% air with 5% CO_2_ at a constant temperature of 37 °C. Sun‐R cells were obtained by constant incubation with low concentrations of sunitinib according to a previous report [5]. In brief, these two cells were exposed to low doses of sunitinib for 3 days and then replaced with fresh media without sunitinib for 24 h. If cells were tolerated at a specific sunitinib concentration, they were subsequently exposed to a higher concentration (0.5 μmol more than previously). If the increased concentration was not tolerated, the cells were maintained in the last sunitinib concentration. The cells were treated for at least 20 passages and then frozen.

### Cell viability assay

Cell viability assay was conducted as previously described using the CCK‐8 kit (Dojindo, Kumamoto, Japan). In brief, cells were seeded onto 96‐well plates at a density of 2 × 10^3^ cells for 24 h, then different concentrations of sunitinib were added for 72 h of treatment, with CCK‐8 reagent added and allowed to incubate at 37 °C in the cell incubator for 2 h. After that, the plate was read at 450 nm using a microplate reader (Life Techniques, Carlsbad, CA, USA).

### Cell apoptosis assay

The detection of cell apoptosis was conducted using flow cytometry. After treatments, RCC cells were digested with trypsin and washed with PBS twice; then cells were incubated with Annexin V and propidium iodide (PI) for 30 min at room temperature. Subsequently, flow cytometry was performed, and the statistical diagrams were drawn by flowjo 7.5 (Ashland, OR, USA).

### Mitochondrial function measurements

The measurements of mitochondrial functions included the detections of mitochondrial respiratory functions by high‐resolution respirometry, detection of anaerobic glycolysis by ECAR and lactate secretions, ROS measurements by specific dyes DHE and MitoSOX (Invitrogen, Thermo Fisher Scientific, Inc.), membrane potentials, NADPH/NADP^+^ ratio and GSH/GSSG ratio (Beyotime Biotechnology, Shanghai, China) according to the protocols of the manufacturers. In brief, high‐resolution mitochondrial respirometry was analyzed using the two‐channel titration injection respirometer Oxygraph‐2k (Oroboros Instruments, Innsbruck, Austria). The parameters of routine respiration (R), CI_OXPHOS_, CI+II_OXPHOS_, CI+II_ETS_ and complex II supported noncoupled respiration, residual oxygen consumption, CIV_ETS_, ECAR and lactate secretions were measured according to previous protocols [43, 44]. Meanwhile, for the detection of ROS, RCC cells were incubated with 5 μm DHE/MitoSOX diluted with DMEM culture medium at 37 °C for 30 min; then the *F*_510 nm_/*F*_580 nm_ ratio was measured using the Fluoroskan Ascent Fluorometer (Life Techniques).

For the measurements of IDH2 activities, Flag‐tagged IDH2 were overexpressed in HEK293T cells, immunoprecipitated and eluted by 250 mg·mL^−1^ Flag peptide (Gilson Biochemical, Madison, WI, USA); then the changes of NADPH fluorescence were measured.

### Quantitative RT‐PCR

Total RNAs were extracted from RCC cells after different treatments. Reverse transcription was performed on 2 μg total RNA by M‐MLV transcriptase (Takara, Kusatsu, Japan). After obtaining the cDNA, qPCR was carried out in triplicate using an ABI 7500 Fast instrument (Applied Biosystems, Foster, CA, USA). The primers used are as follows: Sirt5, forward 5′‐GCACAGAGCCTCGCCTT‐3′, reverse 5′‐GTTGTCGACGACGAGCG‐3′; glyceraldehyde‐3 phosphate dehydrogenase, forward 5′‐TCTCTGCTCCTCCTGTTC‐3′, reverse 5′‐GTTGACTCCGACCTTCAC‐3′.

### Western blotting

Western blotting was conducted as previously described [45, 46]. The primary antibodies used are as follows: anti‐Sirt3 (#5490; Cell Signaling Technology, Boston, MA, USA), anti‐Sirt4 (ab124521; Abcam, Cambridge, MA), anti‐Sirt5 (#8782; Cell Signaling Technology), anti‐α‐tubulin (Beyotime, Shanghai, China), anti‐hexokinase II (ab104836; Abcam), anti‐PDHE1α (ab168379; Abcam), anti‐SDH (ab14714; Abcam), anti‐COX‐IV (ab33985; Abcam), pan‐succinyl‐lysine (PTM‐401), pan‐malonyl‐lysine (PTM‐901), pan‐glutaryl‐lysine (PTM‐1151), pan‐acetyl‐lysine (#9441; Cell Signaling Technology) and Flag (4110‐20; Shanghai Genomics, Shanghai, China). The antibodies were purchased commercially.

### Protein stability assay

Protein stability assay was conducted as previously described [47]. In brief, RCC cells were treated with CHX in a time‐dependent manner to analyze the expression of target proteins. Meanwhile, MG132 was added to block the functions of proteasome; then the expression levels of target protein were analyzed by western blotting.

### Statistical analysis

All data were presented as mean ± standard error of the mean (SEM). All data shown represent the results obtained from at least triplicated independent experiments. Statistical analyses were performed with a two‐tailed unpaired Student's *t*‐test between two groups and with one‐way ANOVA followed by Bonferroni’s *post hoc* tests among three or more groups using graphpad prism version 6.0 ( GraphPad, La Jolla, CA, USA). A *P*‐value <0.05 was considered as statistically significant.

## Conflict of interest

The authors declare no conflict of interest.

## Author contributions

DC and LM conceptualized the idea. LM and GM performed the experiments, analyzed the data and conducted the statistical analysis. LM, GM and LL contributed to the software, experimental methods and interpretation of the data. CH contributed to the Discussion section. LM wrote the draft of the manuscript, while DC revised the manuscript and supervised the study. All authors have read and approved the final version of the manuscript.

## Data Availability

The data will be available from the corresponding author upon reasonable request.
